# Selective Enhancement of the Cell-Permeabilizing Activity of Adenylate Cyclase Toxin Does Not Increase Virulence of *Bordetella pertussis*

**DOI:** 10.3390/ijms222111655

**Published:** 2021-10-28

**Authors:** Jana Holubova, Attila Juhasz, Jiri Masin, Ondrej Stanek, David Jurnecka, Adriana Osickova, Peter Sebo, Radim Osicka

**Affiliations:** 1 Institute of Microbiology of the Czech Academy of Sciences, Videnska 1083, 142 20 Prague 4, Czech Republic; hejnova@biomed.cas.cz (J.H.); attila.juhasz@img.cas.cz (A.J.); masin@biomed.cas.cz (J.M.); stanek@biomed.cas.cz (O.S.); david.jurnecka@biomed.cas.cz (D.J.); osickova@biomed.cas.cz (A.O.); sebo@biomed.cas.cz (P.S.); 2 Czech Centre for Phenogenomics, Institute of Molecular Genetics of the Czech Academy of Sciences, Prumyslova 595, 252 50 Vestec, Czech Republic

**Keywords:** adenylate cyclase toxin, *Bordetella pertussis*, cAMP intoxication, lung colonization, lung inflammation, pore-forming activity, RTX toxin, virulence

## Abstract

The whooping cough agent, *Bordetella pertussis*, secretes an adenylate cyclase toxin–hemolysin (CyaA, ACT, or AC-Hly) that catalyzes the conversion of intracellular ATP to cAMP and through its signaling annihilates the bactericidal activities of host sentinel phagocytes. In parallel, CyaA permeabilizes host cells by the formation of cation-selective membrane pores that account for the hemolytic activity of CyaA. The pore-forming activity contributes to the overall cytotoxic effect of CyaA in vitro, and it has previously been proposed to synergize with the cAMP-elevating activity in conferring full virulence on *B. pertussis* in the mouse model of pneumonic infection. CyaA primarily targets myeloid phagocytes through binding of their complement receptor 3 (CR3, integrin α_M_β_2_, or CD11b/CD18). However, with a reduced efficacy, the toxin can promiscuously penetrate and permeabilize the cell membrane of a variety of non-myeloid cells that lack CR3 on the cell surface, including airway epithelial cells or erythrocytes, and detectably intoxicates them by cAMP. Here, we used CyaA variants with strongly and selectively enhanced or reduced pore-forming activity that, at the same time, exhibited a full capacity to elevate cAMP concentrations in both CR3-expressing and CR3-non-expressing target cells. Using *B. pertussis* mutants secreting such CyaA variants, we show that a selective enhancement of the cell-permeabilizing activity of CyaA does not increase the overall virulence and lethality of pneumonic *B. pertussis* infection of mice any further. In turn, a reduction of the cell-permeabilizing activity of CyaA did not reduce *B. pertussis* virulence any importantly. These results suggest that the phagocyte-paralyzing cAMP-elevating capacity of CyaA prevails over the cell-permeabilizing activity of CyaA that appears to play an auxiliary role in the biological activity of the CyaA toxin in the course of *B. pertussis* infections in vivo.

## 1. Introduction

The Gram-negative coccobacillus *Bordetella pertussis* causes a highly contagious respiratory disease called pertussis, or whooping cough. Pertussis affects children and adults of all age groups and it used to be the major cause of infant mortality before the global introduction of whole cell-based pertussis vaccines seven decades ago [1,2,3]. Unrecognized infections and/or mild disease due to *B. pertussis* continue to persist also in highly vaccinated populations, which makes whooping cough the least controlled vaccine-preventable infectious disease. It is estimated that more than 24 million pertussis cases and ~160,000 pertussis-related deaths occur annually worldwide [4].

The adenylate cyclase toxin (CyaA) belongs to the key virulence factors produced by *B. pertussis* and plays an important role in the early stages of respiratory tract infection [1,2,5,6,7,8,9]. CyaA is a 1706 residues-long polypeptide consisting of an N-terminal adenylate cyclase (AC) enzyme domain (~384 residues) and a pore-forming RTX (Repeats in ToXin) hemolysin (Hly) moiety (~1322 residues) [8,10,11,12]. The Hly comprises (i) a hydrophobic pore-forming domain consisting of residues 500 to 700 [13,14,15,16]; (ii) a fatty acyl-modified segment located between residues 800 to 1000, where the CyaC-mediated activation of proCyaA to CyaA takes place by covalent post-translational acylation of ε-amino groups of lysine residues 860 (K860) and 983 (K983) [17,18]; (iii) a typical calcium-binding RTX domain, harboring the conserved nonapeptide repeats of a consensus sequence X-(L/I/F)-X-G-G-X-G-(N/D)-D, which forms ~40 calcium-binding sites [19], and (iv) a carboxy-proximal secretion signal [11,20,21].

The hemolysin moiety inserts into cellular membranes and mediates translocation of the AC domain directly into the cell cytosol, where the AC is activated by the binding of calmodulin and catalyzes unregulated conversion of ATP to cAMP, thereby subverting cellular signaling [8,12,22]. In parallel, the RTX hemolysin moiety can form small cation-selective membrane pores with a diameter of 0.6 to 0.8 nm, which permeabilize the cellular membrane for potassium ion efflux and can provoke colloid–osmotic (oncotic) cell lysis [13,23]. This cell-permeabilizing activity then synergizes with the cytotoxic effects of elevated cAMP concentrations and contributes to the overall cytotoxicity of CyaA on target cells in vitro [24,25]. While promiscuously acting with a detectable activity on various eukaryotic cells, the CyaA toxin was shown to bind with high affinity and selectivity the CD11b subunit of the CD11b/CD18 heterodimer, known as the α_M_β_2_ integrin, or the complement receptor 3 (CR3) of myeloid phagocytic cells, such as neutrophils, macrophages, or dendritic cells [26,27,28,29,30,31]. CR3 binding enables CyaA to efficiently intoxicate myeloid phagocytes by cAMP. Signaling of cAMP then ablates the oxidative burst and complement-mediated opsonophagocytic bactericidal activities of phagocytes and triggers macrophage apoptosis and/or de-differentiation into monocyte-like cells already at less than 50 pM CyaA concentrations [30,32,33,34,35,36,37,38,39,40].

CyaA-deficient clinical isolates of *B. pertussis* have never been reported and the capacity of CyaA to increase cAMP levels in host sentinel phagocytes was proposed to play a key role in the capacity of *B. pertussis* to proliferate in mouse lungs in the early phases of infection [6,7,41]. However, we recently found that the cAMP elevating capacity of the secreted CyaA toxin was less important for the proliferation of *B. pertussis* in infected mouse lungs than initially claimed [6], once lung colonization by the strains secreting enzymatically active CyaA and inactive CyaA-AC^−^ proteins was compared at equal inoculation dose of 10^5^ CFU [42]. In contrast, the specific role and contribution of the pore-forming activity of CyaA to *B. pertussis* virulence could not be addressed for a long time. Mutants elevating cAMP in cells, but not forming the hemolytic pores, were not available until we constructed the CyaA-E570Q+K860R toxin variant. In this toxin, the cell-invasive AC toxin activity was dissociated from the pore-forming cytolysin activity of CyaA [43]. Due to the E570Q substitution in the pore-forming domain and the K860R substitution ablating the posttranslational acylation of the K860 residue, the CyaA-E570Q+K860R toxin was unable to permeabilize the cellular membrane of CR3-expressing (CR3^+^) phagocytes. In parallel, the specific hemolytic activity of the CyaA-E570Q+K860R toxin on CR3-non-expressing (CR3^−^) erythrocytes was about 70-fold reduced compared to intact CyaA [43]. Nevertheless, the non-hemolytic CyaA-E570Q+K860R toxin still exhibited a full capacity to penetrate the membrane of CR3^+^ cells and intoxicate them by cAMP elevation, whereas its specific capacity to penetrate the membrane of CR3^−^ cells, such as erythrocytes, was about 20-fold lower than that of intact CyaA [43]. Intriguingly, the *B. pertussis* mutant secreting the CyaA-E570Q+K860R toxin variant was non-hemolytic on blood agar plates, but proliferated in infected mouse lungs as efficiently as the hemolytic wild-type *B. pertussis* that secretes the intact CyaA [42]. Importantly, the *B. pertussis* mutant secreting CyaA-E570Q+K860R exhibited a strongly decreased virulence in the pneumonic mouse infection model. It failed to cause a lethal infection even when applied at 10-times higher inoculation doses than is the LD_50_ dose of the wild-type bacteria [42]. Moreover, at an equal bacterial burden in the infected lungs, the *B. pertussis* mutant secreting CyaA-E570Q+K860R elicited a significantly lower level of inflammatory pathology than the parental *B. pertussis* bacteria [42]. This led us to propose that the cell-permeabilizing (hemolytic) activity of CyaA may be required for full virulence of *B. pertussis* infections. Nevertheless, a caveat remained, since the CyaA-E570Q+K860R toxin exhibited a very low capacity to elevate cAMP concentrations in non-myeloid CR3^−^ cells. Hence, it could not be ruled out that cAMP intoxication of the CR3^−^ airway epithelial cells by the intact CyaA, rather than the cell-permeabilizing activity of CyaA, accounted for most of the pro-inflammatory signaling of infected epithelia and the resulting virulence of *B. pertussis* infection. The non-hemolytic mutant secreting the CyaA-E570Q+K860R toxin would not trigger such signaling and the reduction of its virulence could thus not be clearly attributed to the absence of cell-permeabilizing activity of CyaA.

Here, we describe CyaA variants with either selectively and strongly enhanced, or reduced cell-permeabilizing activity, that still exhibit a full capacity to elevate cAMP levels also in CR3^−^ cells, like intact CyaA. Using *B. pertussis* mutants secreting such CyaA variants, we show that the selective enhancement of the cell-permeabilizing capacity of the secreted CyaA toxin does not further increase the virulence and mouse lung colonization capacity of *B. pertussis*.

## 2. Results and Discussion

### 2.1. CyaA Variants with Selectively Up- or Down-Modulated Pore-Forming (Hemolytic) Activity Retain Full cAMP-Elevating Capacity When Secreted from B. pertussis

To assess the impact of the pore-forming activity of CyaA on the virulence of *B. pertussis* in mice, we used two previously described recombinant CyaA variants that exhibit an intact cAMP-elevating capacity on both CR3^+^ and CR3^−^ cells, while exhibiting a selectively enhanced or reduced specific pore-forming (hemolytic) activity [44,45]. The ‘hyperhemolytic’ CyaA-D445N+D446N+E448Q (CyaA-NNQ) mutant produced as a recombinant CyaC-activated protein in *E. coli* was previously found to exhibit a several times higher specific pore-forming activity on both CR3^−^ and CR3^+^ cells than the intact CyaA [44]. In contrast, the recombinant CyaA-V695P variant showed about five times reduced specific pore-forming activity on CR3^−^ cells compared to intact CyaA [45]. Therefore, we introduced the corresponding mutations into the *cyaA* allele on the chromosome of the *B. pertussis* Tohama I strain by marker-less allelic exchange [46].

**Figure 1:**
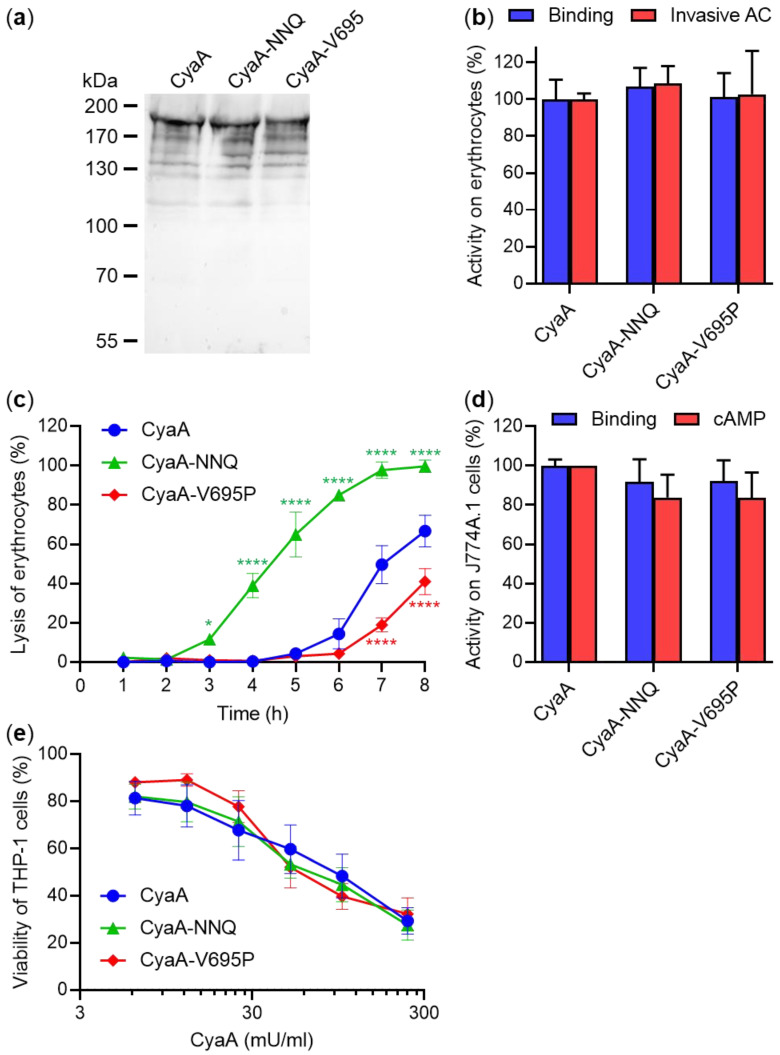
CyaA variants with selectively increased or decreased pore-forming (hemolytic) activity retain full cAMP-elevating capacity when secreted from *B. pertussis*. (**a**) *B. pertussis* Tohama I and its mutant variants were grown in liquid SS medium for 20 h at 37 °C and the CyaA toxin variants were detected on a Western blot of bacterial lysates using the 9D4 monoclonal antibody recognizing the C-terminal RTX domain of CyaA. (**b**) Sheep erythrocytes (5 × 10^8^/mL) were incubated at 37 °C with 0.2 U/mL of the CyaA variants purified on calmodulin-Sepharose beads and after 30 min, aliquots were taken for determination of the cell-associated AC enzyme activity (binding) and of the AC enzyme activity internalized into erythrocytes (invasive AC). The activity of intact CyaA was taken as 100%. Each bar represents the mean value with the standard deviation (SD) of three (binding) or four (invasive AC) independent experiments (*p*-value > 0.05; ANOVA). (**c**) Hemolytic activity was measured upon incubation of sheep erythrocytes with 0.2 U/mL of the CyaA variants as the amount of released hemoglobin by photometric determination (A_541_). Each point represents the mean value ± SD of at least three independent experiments. Significant differences between the mean values of hemolytic activities of intact CyaA and its mutant variants are shown (* *p* < 0.05; **** *p* < 0.0001; ANOVA). (**d**) Binding of the CyaA variants to J774A.1 cells (1 × 10^6^) was determined as the amount of cell-associated AC enzyme activity upon incubation of cells with 0.2 U/mL of the CyaA variants for 30 min at 4 °C. cAMP intoxication was assessed by determining the intracellular concentration of cAMP generated in cells after 30 min of incubation of J774A.1 cells (1.5 × 10^5^) with 32, 16, and 8 mU/mL of the CyaA variants. The activity of intact CyaA was taken as 100%. Each bar represents the mean value with SD of three independent experiments (*p*-value > 0.05; ANOVA). (**e**) Cell viability of CyaA-treated THP-1 cells (1.5 × 10^5^/well) was determined as the capacity of mitochondrial dehydrogenases to reduce the tetrazolium salt WST-1 to its formazan product after 2 h at 37 °C. Each point represents the mean value ± SD of at least three independent experiments (*p*-value > 0.05; ANOVA).

It was important to duly characterize the activities of the here studied toxin variants produced by *B. pertussis* (*Bp*-CyaA, Figure 1a), since the previously characterized recombinant toxin variants (*rEc*-CyaA) were extracted from *E. coli* inclusion bodies with 8 M urea and purified by ion exchange chromatography. In contrast, in *B. pertussis* the toxin is secreted by the type 1 secretion system and in the absence of physiological calcium concentrations (2 mM) in the Stainer–Scholte (SS) medium, *Bp*-CyaA aggregates on the surface of the bacterial outer membrane and can thus be extracted with 4 M urea [21,47]. Due to low yields, *Bp*-CyaA is purified by affinity chromatography on calmodulin-Sepharose beads [48]. More importantly perhaps, in *B. pertussis*, proCyaA is posttranslationally activated by CyaC-catalyzed acylation of the K860 and K983 residues by the saturated palmitoyl (C16:0) chains [17,18]. In contrast, in *E. coli*, the *rEc*-CyaA is acylated by a mixture of saturated palmitoyl (C16:0) and unsaturated palmitoleyl (*cis* Δ9 C16:1) chains [45,49,50,51]. Previous work then revealed that attachment of unsaturated C16:1 chains selectively decreases the pore-forming and hemolytic capacity of *rEc*-CyaA in respect to the exclusively C16:0 acylated *Bp*-CyaA, whereas the specific membrane penetration and cAMP-elevating capacities of *rEc*-CyaA and *Bp*-CyaA are the same [18,49,50,51,52].

We thus first characterized the acylation status of the purified CyaA toxin variants by ultrahigh-resolution Fourier transform ion cyclotron resonance mass spectrometry of tryptic digests separated by liquid chromatography [52]. This confirmed that all three CyaA proteins were acylated exclusively by the palmitoyl chains and, to a very similar extent, on the K860 and K983 residues (Table 1).

For comparison with previous work [44,45], the toxin activities of the CyaA variants were assessed using sheep erythrocytes as model CR3^−^ cells and mouse J774A.1 monocytes/macrophages as model CR3^+^ cells. As shown in Figure 1b, both CyaA mutant variants exhibited an intact capacity to bind erythrocytes and translocate the AC domain across the erythrocyte membrane in a trypsin protection assay. Compared to intact CyaA, the CyaA-NNQ mutant showed a strongly enhanced specific hemolytic activity, whereas the CyaA-V695P mutant permeabilized erythrocytes with a significantly lower activity than the intact toxin (Figure 1c). Under the used conditions, 0.2 U/mL of intact CyaA lysed ~50% of erythrocytes within 7 h, whereas 0.2 U/mL of the CyaA-V695P protein lysed only ~20% of erythrocytes within the same incubation time, and 0.2 U/mL of the CyaA-NNQ toxin reproducibly caused ~50% lysis of erythrocytes already in 4.5 h of incubation (Figure 1c). When tested on CR3^+^ J774A.1 cells, the CyaA-NNQ and CyaA-V695P toxin variants exhibited quite similar binding and cAMP-elevating capacity as the intact CyaA (Figure 1d). Hence, the intact CyaA and the CyaA-NNQ and CyaA-V695P toxin variants exhibited a very similar capacity to produce the cAMP-induced loss of viability of human CR3^+^ THP-1 monocytes (Figure 1e).

All these data show that the CyaA-NNQ and CyaA-V695P mutants secreted by *B. pertussis* bound to and delivered the AC domain into both CR3^−^ and CR3^+^ cells with the same efficacy as intact CyaA, but differ greatly in their specific hemolytic activities. The CyaA-NNQ mutant exhibited a strongly enhanced capacity to permeabilize the target cell membrane, whereas the pore-forming activity of the CyaA-V695P mutant was significantly reduced. Hence, the fact that in *B. pertussis* the CyaA proteins were exclusively acylated by the saturated C16:0 acyl residues did not alter any importantly the relative toxin activities found previously with the *E. coli*-produced *rEc*-CyaA toxin variants mostly acylated by a mixture of C16:0 and C16:1 acyl residues (Table 1).

### 2.2. Selective Increase or Down-Modulation of the Specific Pore-Forming Activity of CyaA Does Not Affect the Lung Colonization Capacity and Virulence of B. pertussis

**Figure 2:**
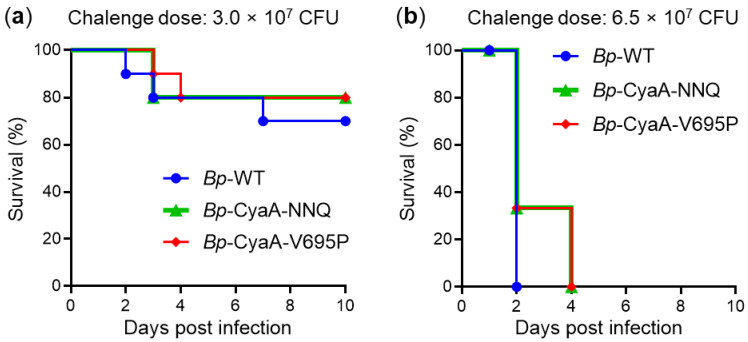
Selective enhancement of the pore-forming activity of CyaA has no impact on the lethality of *B. pertussis* infection. Survival rates of five-week-old BALB/cByJ mice infected with 3.0 × 10^7^ CFU (**a**) or with 6.5 × 10^7^ CFU (**b**) of the WT and mutant *B. pertussis* strains. Mice survival was monitored for 10 days following infection and percentage survival values were calculated using 10 (**a**) or 6 (**b**) mice per challenged group. One representative experiment out of two performed is shown. No significant differences were observed among the survival curves within each challenge group (ANOVA).

To assess the impact of the selectively enhanced or reduced hemolytic (pore-forming) capacity of the secreted CyaA variants on the virulence of *B. pertussis* strains that produce them, we first determined their 50% lethal dose (LD_50_) values using the mouse intranasal challenge model. BALB/cByJ mice were intranasally inoculated with 50 µL of serially diluted bacterial suspensions of the parental *B. pertussis* strain (*Bp*-WT), or its mutant derivatives *Bp*-CyaA-NNQ and *Bp*-CyaA-V695P, respectively, and the survival of infected animals was monitored over 10 days. The experiment was repeated twice, and representative results obtained at two bacterial challenge doses are shown in Figure 2.

At the challenge dose of 3 × 10^7^ colony forming units (CFU), approximately 20% of the infected mice died within 3 (*Bp*-WT and *Bp*-CyaA-NNQ) or 4 (*Bp*-CyaA-V695P) days from intranasal infection (Figure 2a). An additional 10% of mice challenged with *Bp*-WT died on day 7. When an increased challenge dose of 6.5 × 10^7^ CFU was administered, all infected mice died within 2 (*Bp*-WT) or 4 (*Bp*-CyaA-NNQ and *Bp*-CyaA-V695P) days (Figure 2b). The calculated LD_50_ values for *Bp*-WT (~3 × 10^7^ CFU/mouse), *Bp*-CyaA-NNQ (~1.8 × 10^7^ CFU/mouse), and *Bp*-CyaA-V695P (~2.6 × 10^7^ CFU/mouse) were not statistically significantly different. Hence, the selective increase or decrease of the specific pore-forming activity of the secreted CyaA variants did not affect the overall virulence of *B. pertussis* in the mouse pneumonic infection model any importantly, and the mutants secreting the CyaA-NNQ and CyaA-V695P variants caused a comparably lethal course of infection in mice.

**Figure 3:**
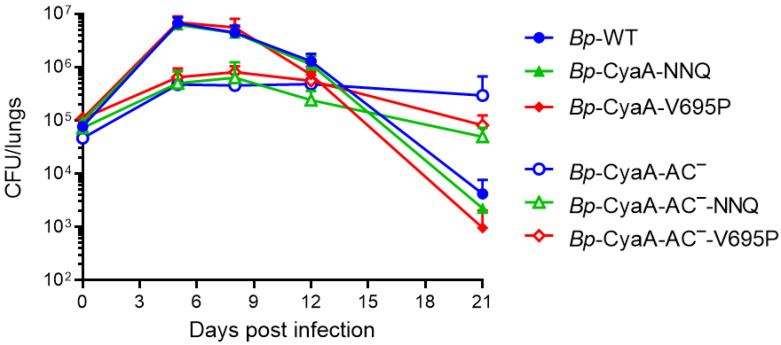
Selective increase of the pore-forming activity of CyaA has no effect on *B. pertussis* lung colonization. Five-week-old BALB/cByJ mice were challenged intranasally with ~1 × 10^5^ CFU of the WT and mutant *B. pertussis* strains. Each point represents the mean value with SD from 3 to 6 mice. No significant differences in lung colonization were observed at any time point within the group of *B. pertussis* strains secreting enzymatically active CyaA toxin variants (*Bp*-WT, *Bp*-CyaA-NNQ, and *Bp*-CyaA-V695P). Similarly, no significant differences in colonization were found within the group of *B. pertussis* strains producing enzymatically inactive CyaA-AC^−^ toxoid variants (*Bp*-CyaA-AC^−^, *Bp*-CyaA-AC^−^-NNQ, and *Bp*-CyaA-AC^−^-V695P). In contrast, significant differences in lung colonization (*p* < 0.0001; ANOVA) were observed between *B. pertussis* strains secreting the CyaA variants and the strains producing the CyaA-AC^−^ variants at days 5 and 8.

To corroborate this observation, the capacity of the mutants to proliferate in mouse lungs was compared to that of the parental *B. pertussis* strain using a sublethal infection dose. BALB/cByJ mice were intranasally inoculated with 50 µL of suspensions containing ~1 × 10^5^ CFU/mouse of *Bp*-WT or its mutant derivatives *Bp*-CyaA-NNQ and *Bp*-CyaA-V695P and lung colonization course was followed for 21 days. Mice were sacrificed by cervical dislocation at 2 h and 5, 8, 12, and 21 days after infection, the lungs were aseptically removed, homogenized in physiological salt solution, and serial dilutions of lung homogenates were plated on Bordet–Gengou agar plates for *B. pertussis* CFU determination after 72 h of incubation at 37 °C. As shown in Figure 3, the *Bp*-CyaA-NNQ and *Bp*-CyaA-V695P mutant strains colonized the lungs of mice as efficiently as the parental *Bp*-WT strain, exhibiting an increase in CFU counts in the lungs by almost two orders of magnitude at the peak of infection 5 days after challenge (~7 × 10^6^ CFU/mouse). The CFU counts of all three *B. pertussis* strains then gradually decreased with a similar time course to a value of ~1 × 10^3^–4 × 10^4^ CFU/mouse on day 21. These results demonstrated that despite a substantial difference in the specific pore-forming activity of the secreted CyaA toxin, the CyaA-NNQ and CyaA-V695P variants supported the capacity of *B. pertussis* to multiply and persist in mouse lungs to the same extent as intact CyaA.

The in vitro cytotoxicity of CyaA action on CR3^+^ phagocytic cells was found to be mainly due to the toxin-catalyzed conversion of cellular ATP to cAMP, which causes BimEL stabilization, Bax activation, and mitochondrial outer membrane permeabilization already at CyaA concentrations as low as 10 ng/mL [38]. This cytotoxic action of CyaA would prevail over the cytotoxic effects of the reduced or enhanced pore-forming activity of the CyaA variants that come in play at orders of magnitude higher CyaA concentrations above ~1 μg/mL [25]. To test this hypothesis, we constructed a set of *B. pertussis* mutants secreting the CyaA-AC^−^ toxoids that were unable to convert ATP to cAMP due to a dipeptide insert between residues 188 and 189, which disrupts the catalytic activity of the AC enzyme [42]. This allowed us to assess the net contribution of the altered pore-forming activities to lung colonization capacities of the *B. pertussis* mutants. As shown in Figure 3, such *B. pertussis* mutants secreting the CyaA-AC^−^ toxoids proliferated in the infected lungs by only one order of magnitude over the inoculation dose. This likely reflected their deficiency in CyaA-mediated disarming of immune cells infiltrating into the infected tissue, such as the inhibition of oxidative burst and of complement-mediated opsonophagocytic killing of bacteria by host neutrophils and macrophages, respectively [30,32,36,37,53]. Moreover, the course of lung infection by the *Bp*-CyaA-AC^−^-NNQ and *Bp*-CyaA-AC^−^-V695P mutant strains secreting the toxoid variants was very similar (Figure 3). Hence, no important impact on the capacity of *B. pertussis* to multiply and persist in mouse lungs was observed with the mutants secreting CyaA-AC^−^ toxoids that exhibited a selectively and importantly enhanced or reduced specific pore-forming capacity. This implies that the pore-forming hemolysin capacity and host cell permeabilization resulting from CyaA toxin action play only an auxiliary role in the contribution of CyaA to the in vivo virulence of *B. pertussis,* which appears to depend exclusively on the capacity of CyaA to intoxicate host cells by elevated cAMP concentrations.

Intriguingly, the *B. pertussis* mutants secreting the CyaA-AC^−^ toxoids exhibited steady ~10^6^ CFU/lung levels on days 5, 8, and 12 post challenge and persisted at higher CFU levels for a longer time than the wild-type *B. pertussis*, reaching a level of ~5 × 10^4^–3 × 10^5^ CFU/lung on day 21 (Figure 3). This surprising phenotype of a persistent infection by the *Bp*-CyaA-AC^−^ bacteria was likely due to a lower level of harnessing of innate immune defense mechanisms by the lower numbers of the *Bp*-CyaA-AC^−^ bacteria producing also lower levels of TLR ligands, making the infection more ‘complaisant’ and chronic. Moreover, the cAMP-intoxicating capacity of the CyaA toxin has previously been shown to elicit the production of interleukin 6 by tracheal epithelial cells, which would activate the cytotoxic action of neutrophils at the site of infection. Furthermore, CyaA-produced cAMP signaling was shown to induce cyclooxygenase 2 expression in macrophages, leading to release of prostaglandins that serve in chemoattraction of neutrophils [7,41,54,55]. It is thus plausible to assume that collateral activation of the innate defenses by the effects of CyaA-produced cAMP signaling would yield high neutrophil recruitment and rapid killing of invading bacteria in the infected lung tissue, once the peak of infection was reached on day 8, resulting in rapid decline of the CFU counts of *B. pertussis* strains producing the active CyaA toxin variants, as documented in Figure 3.

### 2.3. Selective Increase or Decrease of the Specific Pore-Forming Activity of CyaA Does Not Alter the Inflammatory Pathology of B. pertussis-Infected Lungs

**Figure 4:**
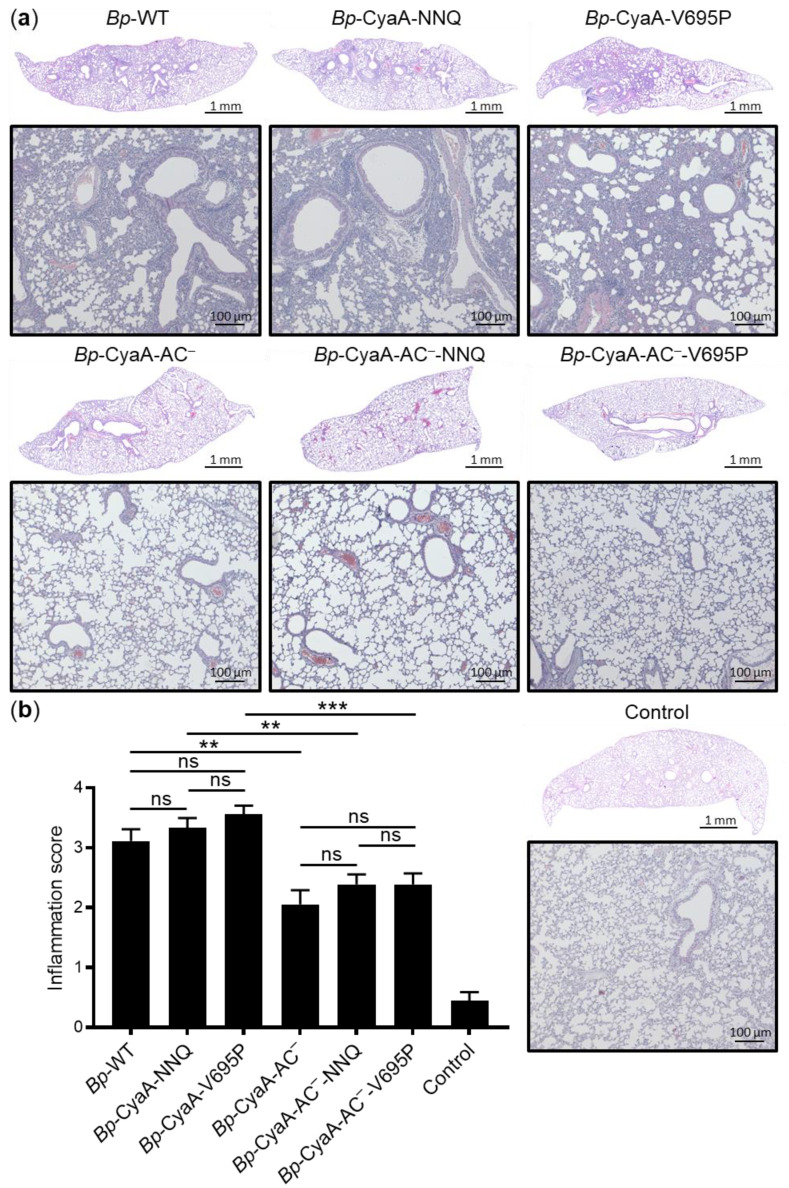
Selective increase of the pore-forming activity of CyaA has no effect on pathology and inflammation of *B. pertussis* infected lungs. Three BALB/cByJ mice per group were infected intranasally with 2 × 10^5^ CFU of the WT and mutant *B. pertussis* strains. Mice were sacrificed on day 7 and lung sections were processed and stained with H&E. Control mice received SS medium only. (**a**) Scanned longitudinal sections of the left lobes that are representative of six serial sections per lung lobe. The bottom panels show enlargements of representative images of the bronchi and peribronchial parenchyma taken by a light microscope at a magnification of 10×. Lungs of mice infected by the group of *B. pertussis* strains secreting enzymatically active CyaA toxin variants (*Bp*-WT, *Bp*-CyaA-NNQ and *Bp*-CyaA-V695P) exhibited bronchopneumonia affecting the regions primarily around the large lobar bronchi. Substantially milder inflammation was observed in the lungs of mice infected by the group of *B. pertussis* strains producing enzymatically inactive CyaA-AC^−^ toxoid variants (*Bp*-CyaA-AC^−^, *Bp*-CyaA-AC^−^-NNQ, and *Bp*-CyaA-AC^−^-V695P). (**b**) Quantification of H&E stained slides was performed manually by the inflammation score 0–4 (0—no inflammation; 1—1–25% inflammation; 2—26–50% inflammation; 3—51–75% inflammation; 4—76–100% inflammation) that was calculated as the ratio of the total inflammatory lesion area to the total area of each lung section on 6 consecutive lung sections for 3 infected mice per group (18 sections analyzed in total; ** *p* < 0.01; *** *p* < 0.001; ns, not significant; ANOVA).

Previously, we observed that infection with a *B. pertussis* strain secreting a CyaA-E570Q+K860R toxin variant capable to intoxicate by cAMP only CR3^+^ myeloid cells and not CR3^−^ cells caused a significantly lower inflammatory pathology in infected mouse lungs than wild-type *B. pertussis,* despite an equal capacity to proliferate in the lungs [42]. Therefore, we investigated the extent of lung pathology and inflammation provoked by infections with the *B. pertussis* strains that secreted CyaA toxin variants exhibiting a selectively enhanced or reduced pore-forming capacity, but capable to intoxicate by cAMP elevation both CR3^+^ and CR3^−^ cell types. BALB/cByJ mice were intranasally infected with ~2 × 10^5^ CFU of bacterial suspensions and sacrificed on day 7. Formalin-fixed lungs of infected animals were processed for histological examination, and six representative 2-µm thick sections of the left lobe of each sample set were cut every 150 µm and stained with hematoxylin and eosin (H&E). Pathological changes in the H&E stained lung sections were assessed (Figure 4a) and quantified using an inflammation score ranging from 0 to 4 (Figure 4b) by an experienced pathologist in a blinded manner.

Using this scoring, the lungs of control mice inoculated with SS medium yielded an inflammation score of less than 0.5 (Figure 4b) and exhibited an unaltered microscopic structure with continuous alveolar septa, intact bronchi and bronchioles, and well-preserved respiratory epithelium (Figure 4a). In contrast, the lungs of mice inoculated with *Bp*-WT and its mutant derivatives *Bp*-CyaA-NNQ and *Bp*-CyaA-V695P exhibited a high level of inflammation with conspicuous bronchopneumonic lesions that spread peribronchially with involvement of the peribronchial parenchyma (Figure 4a). The inflammatory cellular infiltrates consisted predominantly of polymorphonuclear granulocytes, substantially higher numbers of monocytes/macrophages, and swollen alveolar cells. The bronchial respiratory epithelium showed a characteristic focal to discrete hypertrophy and marked congestion in the swollen pulmonary regions. Quantification of the inflammation score revealed that the infection with the mutant strains *Bp*-CyaA-NNQ (score 3.3 ± 0.7) and *Bp*-CyaA-V695P (score 3.6 ± 0.6) elicited a comparable level of inflammatory pathology in mouse lungs as the parental *Bp*-WT infections (score 3.1 ± 0.8) (Figure 4b).

In contrast, as also shown in Figure 4a, the infections of mouse lungs with the *Bp*-CyaA-AC^−^, *Bp*-CyaA-AC^−^-NNQ, and *Bp*-CyaA-AC^−^-V695P mutant strains secreting enzymatically inactive toxoids elicited a distinctly milder lung pathology than the infections with the *B. pertussis* strains secreting the CyaA toxin variants that proliferated in the lungs to about an order of magnitude higher levels (Figure 3). The alveoli in lungs infected with the toxoid-secreting strains remained well preserved, and only occasional focal alveolar pneumonic foci were observed. The peribronchial parenchyma was usually uniform and without signs of hypertrophy. Occasionally, a peribronchial inflammatory infiltrate was present, extending into the vicinity of the bronchi, but rarely affecting the adjacent alveoli. Mild lung congestion was also observed, but it was less pronounced than in the lungs infected with the *B. pertussis* strains secreting the enzymatically active CyaA toxin variants (Figure 4a). Quantification of the inflammation score of the infected lungs revealed that the *Bp*-CyaA-AC^−^-NNQ (score 2.4 ± 0.7) and *Bp*-CyaA-AC^−^-V695P (score 2.4 ± 0.8) mutant strains caused lower inflammatory damage of infected mouse lungs, alike the *Bp*-CyaA-AC^−^ infection (score 2.1 ± 1.0) (Figure 4b).

**Figure 5:**
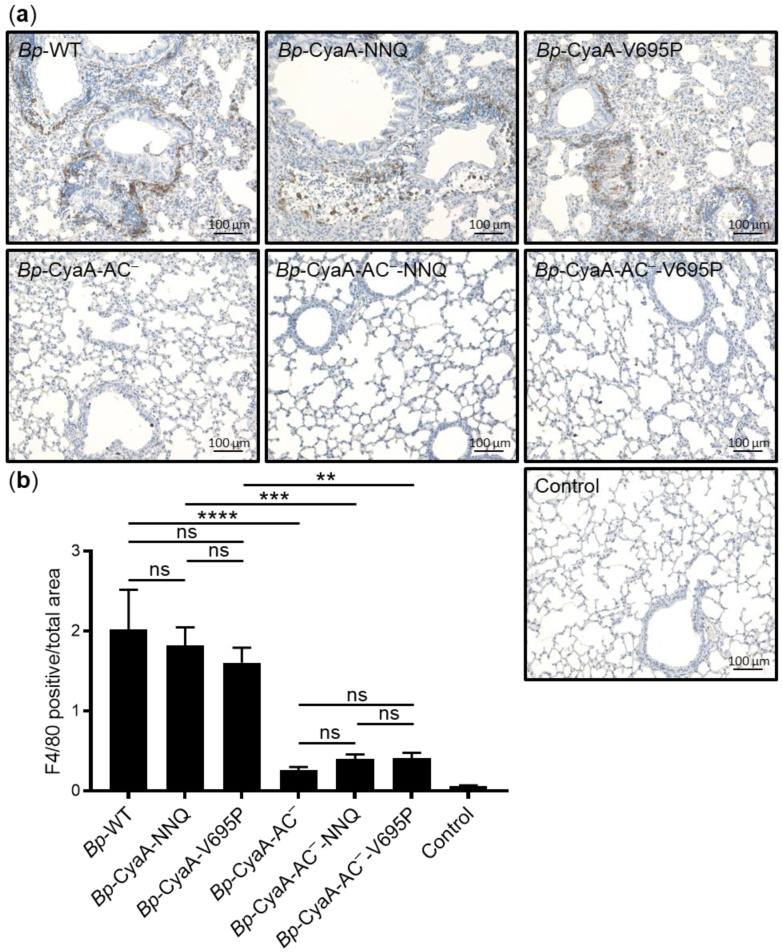
Selective enhancement of the pore-forming activity of CyaA does not affect macrophage recruitment into *B. pertussis* infected lungs. Three BALB/cByJ mice per group infected intranasally with 2 × 10^5^ CFU of the WT and mutant *B. pertussis* strains were sacrificed on day 7 and the sections of lung tissue were examined upon F4/80 immunohistochemical staining for macrophages. (**a**) Representative lung sections were inspected by light microscopy at a magnification of 10× and infiltration of macrophages in the *B. pertussis*-induced lesions was assessed. Substantially lower macrophage infiltration was observed in the lungs of mice infected by the group of *B. pertussis* strains producing enzymatically inactive CyaA-AC^−^ toxoid variants (*Bp*-CyaA-AC^−^, *Bp*-CyaA-AC^−^-NNQ, and *Bp*-CyaA-AC^−^-V695P) than in the lungs of mice infected by *B. pertussis* strains secreting enzymatically active CyaA toxin variants (*Bp*-WT, *Bp*-CyaA-NNQ, and *Bp*-CyaA-V695P). (**b**) Quantification of F4/80-stained macrophages was performed by an automated evaluation for positive pixel count for DAB using the QuPath software on 6 consecutive lung sections for 3 infected animals per group (18 sections analyzed in total; ** *p* < 0.01; *** *p* < 0.001; **** *p* < 0.0001; ns, not significant; ANOVA).

To determine whether the up- or down-modulated specific pore-forming activity of CyaA produced by infecting bacteria affects macrophage recruitment into infected lungs, we assessed the infiltration of macrophages on lung sections by immunostaining for the F4/80 macrophage marker. As shown in Figure 5a, a strongly increased signal corresponding to macrophages was observed in the inflamed parenchyma of lungs infected with *Bp*-WT and its mutant derivatives *Bp*-CyaA-NNQ and *Bp*-CyaA-V695P, compared to the lungs of control mice inoculated with sterile SS medium. Nevertheless, quantification of F4/80-stained macrophages using the QuPath software revealed that the *Bp*-CyaA-NNQ and *Bp*-CyaA-V695P mutant infections elicited recruitment of similar numbers of macrophages into the inflamed lungs as the parental *Bp*-WT strain (Figure 5b). In contrast, the *Bp*-CyaA-AC^−^, *Bp*-CyaA-AC^−^-NNQ, and *Bp*-CyaA-AC^−^-V695P mutant strains, secreting toxoid forms of CyaA and proliferating to about an order of magnitude lower bacterial counts in the lungs, provoked only low level of macrophage infiltration into lung tissue (Figure 5a). Infections by all three toxoid-secreting strains then attracted comparable numbers of macrophages into the lungs (Figure 5b).

In summary, all these results show that a substantial difference in pore-forming activity of the secreted CyaA toxin variants harboring the NNQ or V695P substitutions had no clear impact on the pathology and inflammation elicited by *B. pertussis* infection of mouse lungs. The inflammatory damage was driven by the level of bacterial proliferation in the lungs and depended on the enzymatic capacity of CyaA to intoxicate cells by conversion of ATP to cAMP and not on the cell-permeabilizing activity of the toxin.

## 3. Materials and Methods

### 3.1. Bacterial Strains and Growth Conditions

The *Escherichia coli* strain XL1-Blue was used throughout this work for plasmid construction and the *E. coli* strain SM10 λ pir was used for plasmid transfer into *B. pertussis* by bacterial conjugation. *E. coli* strains were cultivated at 37 °C on Luria–Bertani (LB) agar medium or in LB broth. When appropriate, LB culture media were supplemented with ampicillin (pSS4245 plasmid-carrying transformants, 100 μg/mL). The *B. pertussis* Tohama I strain was obtained from the Institute Pasteur Collection of Cultures (Paris, France) under the catalogue No. CIP 81.32. The parental *B. pertussis* and derived mutant strains were grown on Bordet–Gengou (BG) agar plates (Difco, Franklin Lakes, NJ, USA) supplemented with 1% glycerol, 15% defibrinated sheep blood (LabMediaServis, Jaromer, Czech Republic) at 37 °C in a 5% CO_2_ atmosphere for 72 h to visualize hemolysis. Liquid cultures were obtained by growing bacteria overnight in modified Stainer–Scholte (SS) medium [56] supplemented with 3 g/L of Casamino Acids (Difco, Franklin Lakes, NJ, USA) and 1 g/L of heptakis (2.6-di-O-dimethyl) β-cyclodextrin (Sigma-Aldrich, St. Louis, MO, USA) to a mid-exponential phase (*B. pertussis* OD 600 = 1.0) at 37 °C.

### 3.2. Mutagenesis of the cyaA Gene on B. pertussis Chromosome

Construction of the alleles for production of the CyaA-D445N+D446N+E448Q (CyaA-NNQ) and CyaA-V695P mutant variants, as well as their CyaA-AC^−^ variants (188–GlySer–189) in *E. coli* was described in detail previously [44,45]. The respective mutated *cyaA* gene segments were cloned into the exchange vector plasmid pSS4245 (*ori*V, *Amp*R, *Str*R, *Km*R, *Ble*R, *Tet*R, and an I-*Sce*I cleavage site for counterselection) kindly provided by Dr. Scott Stibitz (U.S. CBER, FDA, MD, USA) and introduced by allelic exchange into the *B. pertussis* chromosome as described previously [42]. For each construct, several individual colonies were characterized for phenotypic change and the presence of introduced mutations and the absence of undesired mutations were verified by sequencing of relevant portions of PCR-amplified segments of the *cyaA* gene.

### 3.3. Western Blotting

The *B. pertussis* strains were grown in liquid SS medium for 18 h at 37 °C and cells from 1 mL of bacterial suspension were collected by centrifugation (10 min, 15,000× *g*). The pellet was resuspended in 100 µL of Laemmli buffer (50 mM Tris-HCl (pH 6.8), 2% SDS, 0.1% bromophenol blue, 10% glycerol, 1% β-mercaptoethanol) and heated for 5 min at 100 °C. Bacterial lysates were separated by 7.5% SDS-polyacrylamide gel electrophoresis (SDS-PAGE) and transferred to a nitrocellulose membrane. The CyaA variants were detected by the 9D4 monoclonal antibody (mAb; at a 1:3000 dilution) [57] and revealed by horseradish peroxidase (HRP)-conjugated secondary antibody (at a 1:10,000 dilution; GE Healthcare, Chicago, IL, USA) using a chemiluminescence detection kit (Thermo Fisher Scientific, Waltham, MA, USA) and an LAS-4000 imaging system instrument (GE Healthcare Bio-Sciences AB, Uppsala, Sweden).

### 3.4. Purification of CyaA Variants from B. pertussis Cultures

*B. pertussis* strains were grown for 72 h on BG agar plates and subcultured in 4 × 500 mL of SS medium to an A_600_ of 2–3. The cultures were collected by centrifugation (30 min at 5150× *g*) and resuspended in 30 mL of 50 mM Tris-HCl (pH 8.0). The cultures were again centrifuged (20 min at 23,880× *g*), the pellets were resuspended in 10 mL of 50 mM Tris-HCl (pH 8.0) and 8 M urea in 50 mM Tris-HCl (pH 8.0) was added dropwise to reach a final 4 M urea concentration. The outer membrane-attached toxin was extracted with 4 M urea for 30 min at 4 °C. The extracts were separated from bacterial cells by centrifugation (20 min at 23,880× *g*), diluted twice in ice-cold washing buffer (50 mM Tris-HCl (pH 8.0), 500 mM NaCl, 2 mM CaCl_2_) and loaded on a calmodulin-Sepharose 4B column (GE Healthcare, Pittsburgh, PA, USA) equilibrated with the same buffer. After washing the column with washing buffer, the CyaA variants were eluted with 50 mM Tris-HCl (pH 8.0), 8 M urea and 2 mM EDTA. Prior to the determination of CyaA activities, all CyaA samples were diluted out from the 100× concentrated toxin stocks in buffered 8 M urea solutions to their final working concentrations by direct 100-fold dilution into cell suspensions in buffers/media containing 2 mM Ca^2+^ ions, which drive the folding of CyaA to biologically active toxin capable to bind and penetrate cellular membranes [25].

### 3.5. Purification of Recombinant CyaA Variants from E. coli Cultures

The recombinant intact CyaA toxin and its mutant variants CyaA-D445N+D446N+E448Q (CyaA-NNQ) and CyaA-V695P were produced in *E. coli* and purified to homogeneity, as previously described [44,45].

### 3.6. Assay of AC Activity

AC enzymatic activity was determined in the presence of 1 µM calmodulin, as previously described [58]. One unit (U) of adenylate cyclase enzyme (toxin) activity corresponds to 1 μmol of cAMP formed in 1 min at pH 8.0 at 30 °C.

### 3.7. Cell Binding, Cell-Invasive and Hemolytic Activities in Sheep Erythrocytes

Sheep erythrocytes (LabMediaServis, Jaromer, Czech Republic) were washed in TNC buffer (50 mM Tris-HCl (pH 7.4), 150 mM NaCl, 2 mM CaCl_2_), adjusted to 5 × 10^8^ cells/mL, and incubated with the purified CyaA variants at 37 °C in TNC buffer. After 30 min, cells were washed in TNC buffer to remove unbound CyaA and divided into two aliquots. One aliquot was after extensive washing in cold TNE buffer (50 mM Tris-HCl (pH 7.4), 150 mM NaCl, 5 mM EDTA) directly used to determine the amount of cell-associated AC activity (binding, membrane-bound CyaA). The other aliquot was treated with 20 µg/mL of trypsin for 15 min at 37 °C to inactivate the extracellular AC enzyme that did not translocate across the cellular membrane. Soybean trypsin inhibitor (40 µg/mL) was then added to the mixture to stop the reaction before the samples were washed twice in cold TNE buffer and used to determine the amount of cell-invasive AC enzyme activity. The activity of intact CyaA was taken as 100%. Hemolytic activity was measured by determining the hemoglobin release (A_541_) upon incubation of the CyaA variants with 5 × 10^8^/mL washed erythrocytes in TNC buffer.

### 3.8. Binding of CyaA to J774A.1 Cells

The CR3-positive mouse J774A.1 macrophages (ATCC TIB 67, 1 × 10^6^/mL) were incubated in Dulbecco’s Modified Eagle Medium (D-MEM) with the CyaA variants for 30 min at 4 °C, prior to removal of unbound CyaA by three washes with D-MEM. Cells were lysed with 0.1% Triton X-100 and the membrane-associated AC enzyme activity was determined as described above.

### 3.9. cAMP Determination

Mouse J774A.1 cells (1 × 10^5^/well) were incubated at 37 °C with the CyaA variants for 30 min in D-MEM. The reaction was stopped by the addition of 0.2% Tween-20 in 100 mM HCl, samples were boiled for 15 min at 100 °C, neutralized by the addition of 150 mM unbuffered imidazole, and cAMP was measured by a competitive immunoassay as previously described [59]. The activity of intact CyaA was taken as 100%.

### 3.10. Cell Viability Assay

Cell viability of CR3-positive THP-1 monocytes (ATCC TIB 202) following exposure to the CyaA variants was determined in D-MEM medium as the capacity of mitochondrial reductases to convert the tetrazolium salt WST-1 (4-[3-(4-iodophenyl)-2-(4-nitrophenyl)-2H-5-tetrazolio]-1,3-benzene disulfonate) to formazan [24,25,60], using the WST-1 assay kit (Roche, Basel, Switzerland) according to the manufacturer’s protocol.

### 3.11. Analysis of the Acylation Pattern of CyaA Variants

The acylation pattern of the purified CyaC-acylated CyaA variants was determined by liquid chromatography-mass spectrometry (LC-MS), as previously described [52,61].

### 3.12. Ethics Statement

All animal experiments were approved (permission No. 41/2019) by the Animal Welfare Committee of the Institute of Molecular Genetics of the Czech Academy of Sciences, v. v. i., Prague, Czech Republic. Handling of animals was performed according to the Guidelines for the Care and Use of Laboratory Animals, the Act of the Czech National Assembly, Collection of Laws no. 246/1992.

### 3.13. Animal Infection Experiments

Five-week-old female BALB/cByJ mice (Charles River, France) were used in this study. Mice were anesthetized by intraperitoneal (i.p.) injection of ketamine (80 mg/kg) and xylazine (8 mg/kg) in saline and were inoculated intranasally with 1 × 10^5^ CFU of *B. pertussis* cell suspensions delivered in 50 µL volume. To determine viable CFU, the suspensions were diluted in PBS and plated on BG agar plates.

To determine *B. pertussis* lung colonization, infected BALB/cByJ mice (3 to 6 mice per time point) were sacrificed by cervical dislocation 2 h after exposure to challenge suspension (day 0) and on the indicated days thereafter (day 5, 8, 12, and 21). Lungs were aseptically removed and homogenized in physiological solution with tissue grinders (Merck, Darmstadt, Germany). Dilutions of lung homogenates were plated on BG agar plates and CFU were counted after 72 h of incubation at 37 °C.

For determination of the LD_50_ values, groups of 6 or 10 mice were challenged intranasally with serially diluted bacterial suspensions 1.0 × 10^7^, 3.0 × 10^7^, or 6.5 × 10^7^ CFU/mouse, and their survival was monitored over 10 days. The results are expressed as the percentage of surviving mice and are representative of two independent experiments. The LD_50_ values were calculated by the probit analysis method of Finney using the freeware LD_50_/LC_50_ calculator downloaded at https://goo.gl/9QYcNk, as described at https://probitanalysis.wordpress.com/2016/07/07/first-blog-post/, (accessed on 12 November 2019).

### 3.14. Histological Studies

Lung morphology was examined 7 days upon the intranasal challenge of mice with a dose of 2 × 10^5^ CFU of *B. pertussis* strains. Control mice were treated with 50 µL of SS medium only. The animals were anesthetized as described above and sacrificed by cervical dislocation. Lungs were removed, fixed in 4% phosphate-buffered formaldehyde for 48 h at 4 °C, and transferred into 70% ethanol for 24 h at 4 °C. The tissue samples were processed in a Leica ASP6025 Tissue Processor (Leica Biosystems, Nussloch, Germany) with an automated protocol and embedded in paraffin blocks. Six representative 2-µm thick sections of the left lobe were cut every 150 µm from each sample using a Leica RM 2255 Microtome (Leica Biosystems, Nussloch, Germany) and placed onto SuperFrost+ slides (Epredia, Kalamazoo, MI, USA).

Hematoxylin and eosin (H&E) staining was performed using an automated Leica ST5020 Staining Machine (Leica Biosystems, Nussloch, Germany) and coverslipping was carried out using a Leica CV5030 Coverslipper (Leica Biosystems, Nussloch, Germany). Quantification of H&E stained slides was performed manually by inflammation score 0–4 (0—no inflammation; 1—1–25% inflammation; 2—26–50% inflammation; 3—51–75% inflammation; 4—76–100% inflammation). It was calculated as the ratio of the total inflammatory lesion area to the total area of each lung section and values calculated for six consecutive lung sections for 3 infected mice per group were averaged and expressed as means with SD (18 sections analyzed in total).

For immunohistochemistry staining for the macrophage marker F4/80, the tissue sections were dried for 1 h at 65 °C, deparaffinized using an automated LEICA ST5020 machine (Leica Biosystems, Nussloch, Germany), and rehydrated. Antigen retrieval was performed with citrate buffer HIER, pH 6.0 (Zytomed, Berlin, Germany) in a pressure cooker for 15 min at 110 °C. Endogenous peroxidase activity was blocked with 3% hydrogen peroxide in methanol (*v*/*v*) for 15 min at room temperature (RT) and 2% BSA (Carl Roth, Karlsruhe, Germany) was used for 20 min at RT to prevent unspecific binding. The tissue sections were then stained with the rabbit anti-F4/80 mAb (Cell Signaling Technology, Danvers, MA, USA) at a 1:800 dilution in Antibody Diluent (Zytomed, Berlin, Germany) overnight at 4 °C, followed by incubation with anti-rabbit HRP-conjugated polymer for 30 min at RT. The immune reaction was developed using 3,3’-diaminobenzidine (DAB) solution (Dako, Glostrup, Denmark) for 10 min at RT in the dark, and counterstaining was performed with hematoxylin H (Biognost, Zagreb, Croatia) for 2 min at RT. Slides were mounted with Aquatex mounting medium (Merck-Millipore, Darmstadt, Germany) and visualized using an AxioScan.Z1 automated slide scanner (Carl Zeiss, Göttingen, Germany) or a light microscope Zeiss Scope A1 (Carl Zeiss, Göttingen, Germany). Quantification of F4/80-stained macrophages was performed by an automated evaluation for positive pixel count for DAB on six consecutive lung sections for 3 infected mice per group (18 sections analyzed in total) using the QuPath Software, v0.2.3 (University of Edinburgh, Edinburgh, UK).

### 3.15. Statistical Analysis

The results are expressed as the arithmetic means ± SD of the mean. Statistical analysis was performed by one-way ANOVA with Tukey multiple comparisons test, by two-way ANOVA with Sidak’s multiple comparisons test, or Log-rank (Mantel–Cox) test using GraphPad Prism 9.1.0 (GraphPad Software, La Jolla, CA, USA).

## 4. Conclusions

We previously constructed a *B. pertussis* strain secreting the CyaA-E570Q+K860R mutant toxin that was almost completely inactive on non-myeloid CR3^−^ cells, such as erythrocytes or airway epithelial cells [43]. However, this toxin variant retained a full capacity to elevate cAMP levels in the cytosol of CR3^+^ cells, despite a selective reduction of its pore-forming activity on CR3^+^ cells by about an order of magnitude, as compared to intact CyaA [42,43]. In the mouse pneumonic infection model, the *Bp*-CyaA-E570Q+K860R mutant strain exhibited an approximately tenfold reduction in virulence, as judged by an increase of LD_50_ from ~4.1 × 10^7^ CFU/mouse of the parental *Bp*-WT strain to LD_50_ of ~3.5 × 10^8^ CFU/mouse, with infected mice dying over a week later than upon infection with the WT strain [42]. This indicated that the pore-forming activity of CyaA and/or its capacity to elevate cAMP in CR3^−^ cells was required for full virulence of *B. pertussis* [42]. However, the used mutant did not allow to exclude that it was rather the contribution of the cAMP elevation in CR3^−^ cells that was missing for full virulence, than the pore-forming activity. Here, we found that *B. pertussis* mutants secreting CyaA capable to intoxicate both CR3^+^ and CR3^−^ cells and exhibiting either a selectively and strongly enhanced or a selectively reduced hemolytic (pore-forming) activity, exhibited equal virulence and mouse lung colonization capacity as the parental *Bp*-WT strain. Since the modulation of the pore-forming activity did not affect the lethality of *B. pertussis* infection, it appears that the full virulence of *B. pertussis* depends predominantly on the capacity of CyaA to elevate cAMP in both myeloid CR3^+^ and non-myeloid CR3^−^ cells, such as airway epithelial cells colonized by *B. pertussis* bacteria.

## Figures and Tables

**Table 1:** Acylation status of the CyaA variants purified from *B. pertussis* and *E. coli*.

Acyl Chain ^1^	*B. pertussis*						*E. coli*					
	CyaA		CyaA-NNQ		CyaA-V695P		CyaA		CyaA-NNQ		CyaA-V695P	
	K860	K983	K860	K983	K860	K983	K860	K983	K860	K983	K860	K983
**None**	-	10	-	17	-	14	9	2	5	2	19	7
**C16:0**	100	90	100	81	100	83	23	40	17	26	20	36
**C16:1**	-	-	-	-	-	-	61	47	75	60	55	49
**C14:0**	-	-	-	2	-	3	3	2	-	1	4	4
**C16:1-OH**	-	-	-	-	-	-	-	1	-	1	-	-
**C18:1**	-	-	-	-	-	-	4	8	3	10	2	4

^1^ The CyaA variants were produced in the presence of the CyaC acyltransferases in *B. pertussis* or *E. coli* cells, purified close to homogeneity and analyzed by MS. Percentage distributions of the acyl chains linked to the ε-amino groups of the residues K860 and K983 were estimated semiquantitatively, from the relative intensities of selected ions in reconstructed ion current chromatograms. The sign “-“ means that the acyl chain was not detected.
